# Supplementary material for: The Validity of Ultra-Short-Term Heart Rate Variability during Cycling Exercise
Source: Sensors (Basel). 2023 Mar 22;23(6):3325. doi: 10.3390/s23063325 (PMC10058535; doi:10.3390/s23063325)
Supplement: Supplementary file 1 [file sensors-23-03325-s001.zip › sensors-2278185-supplementary.pdf]

Supplementary Materials

Table S1. The absolute values of HRV during exercise.

Variable	Exercise intensity	Time segments of HRV analysis				
		180 sec	120 sec	90 sec	60 sec	30 sec
meanRR (ms)	20%VO_{2peak}	752.2 ± 116.4	744.8 ± 113.0	738.8 ± 111.4	729.1 ± 111.5	718.2 ± 102.9
	50%VO_{2peak}	568.0 ± 65.4	548.2 ± 63.0	539.1 ± 62.9	530.4 ± 60.6	522.8 ± 58.9
	80%VO_{2peak}	408.0 ± 45.5	394.9 ± 42.6	388.7 ± 40.9	383.2 ± 39.3	378.4 ± 38.4
HR (beat/min)	20%VO_{2peak}	81.7 ± 13.1	82.4 ± 13.2	83.1 ± 13.4	84.3 ± 13.7	85.3 ± 13.0
	50%VO_{2peak}	107.0 ± 12.3	110.9 ± 12.8	112.8 ± 13.2	114.6 ± 13.1	116.2 ± 13.1
	80%VO_{2peak}	148.7 ± 15.7	153.6 ± 15.6	155.9 ± 15.4	158.1 ± 15.2	160.0 ± 15.2
Time-domain						
SDNN (ms)	20%VO_{2peak}	38.4 ± 17.3	37.3 ± 17.3	36.2 ± 17.6	35.0 ± 17.5	33.0 ± 17.2
	50%VO_{2peak}	13.5 ± 6.2	11.1 ± 5.2	10.5 ± 5.1	9.8 ± 4.6	9.3 ± 5.1
	80%VO_{2peak}	3.3 ± 1.2	2.7 ± 1.0	2.5 ± 0.9	2.4 ± 0.9	2.4 ± 0.9
RMSSD (ms)	20%VO_{2peak}	38.1 ± 23.8	37.3 ± 24.1	36.0 ± 24.6	34.4 ± 24.3	31.6 ± 21.9
	50%VO_{2peak}	10.7 ± 6.1	8.5 ± 4.4	7.6 ± 4.1	7.3 ± 3.6	7.0 ± 4.0
	80%VO_{2peak}	3.2 ± 1.2	2.9 ± 1.1	2.8 ± 1.1	2.8 ± 1.1	2.7 ± 1.2
Frequency-domain						
LF (ms^2)	20%VO_{2peak}	737.3 ± 560.5	714.3 ± 551.3	676.0 ± 518.1	612.4 ± 411.4	572.2 ± 410.6
	50%VO_{2peak}	110.2 ± 86.4	77.6 ± 71.9	78.6 ± 80.9	65.6 ± 71.7	53.4 ± 64.2
	80%VO_{2peak}	5.7 ± 6.2	3.5 ± 5.1	3.4 ± 6.3	2.3 ± 2.9	2.3 ± 3.0
HF (ms^2)	20%VO_{2peak}	490.9 ± 445.6	474.6 ± 442.8	460.2 ± 472.5	423.9 ± 427.0	342.3 ± 352.7
	50%VO_{2peak}	77.6 ± 93.1	52.9 ± 65.7	45.8 ± 53.8	41.6 ± 51.9	44.2 ± 61.5
	80%VO_{2peak}	6.8 ± 9.7	5.1 ± 10.6	5.4 ± 14.2	3.5 ± 5.1	4.7 ± 10.2
LF/HF	20%VO_{2peak}	2.4 ± 1.9	2.6 ± 2.3	2.7 ± 2.8	2.6 ± 3.0	2.5 ± 2.3
	50%VO_{2peak}	2.2 ± 1.4	2.3 ± 1.4	2.6 ± 2.0	2.5 ± 1.8	2.1 ± 1.6
	80%VO_{2peak}	1.4 ± 1.1	1.2 ± 1.0	1.4 ± 1.5	1.6 ± 1.7	2.3 ± 4.5
Heart rate/LF	20%VO_{2peak}	0.2 ± 0.2	0.2 ± 0.2	0.2 ± 0.3	0.3 ± 0.3	0.6 ± 1.2
	50%VO_{2peak}	2.0 ± 2.7	3.0 ± 2.9	5.1 ± 10.5	5.0 ± 8.3	12.1 ± 27.5
	80%VO_{2peak}	116.5 ± 251.6	179.4 ± 247.1	196.2 ± 221.9	235.7 ± 271.8	372.3 ± 434.5
Non-linear analysis						
DFAα1	20%VO_{2peak}	1.04 ± 0.25	1.04 ± 0.25	1.03 ± 0.25	1.08 ± 0.30	-
	50%VO_{2peak}	1.17 ± 0.17	1.18 ± 0.20	1.21 ± 0.18	1.19 ± 0.23	1.21 ± 0.21
	80%VO_{2peak}	0.86 ± 0.28	0.73 ± 0.26	0.72 ± 0.29	0.68 ± 0.31	0.66 ± 0.35
DFAα2	20%VO_{2peak}	0.32 ± 0.16	0.32 ± 0.13	0.32 ± 0.14	0.38 ± 0.18	-
	50%VO_{2peak}	0.55 ± 0.18	0.58 ± 0.19	0.59 ± 0.20	0.54 ± 0.27	0.70 ± 0.28
	80%VO_{2peak}	0.92 ± 0.24	0.92 ± 0.20	0.96 ± 0.23	1.02 ± 0.33	1.01 ± 0.44
SampEn	20%VO_{2peak}	1.74 ± 0.29	1.74 ± 0.35	1.80 ± 0.41	1.87 ± 0.57	-
	50%VO_{2peak}	1.51 ± 0.27	1.55 ± 0.23	1.56 ± 0.21	1.58 ± 0.29	1.61 ± 0.28
	80%VO_{2peak}	1.80 ± 0.19	1.84 ± 0.17	1.80 ± 0.18	1.82 ± 0.28	1.90 ± 0.57

Values are mean ± SD. Note that there were missing data (see Materials and Methods section). HR indicates heart rate; SDNN, standard deviation of all RR intervals; RMSSD, root mean square of successive RR interval differences, LF, low-frequency power; HF, high-frequency power; DFA, detrended fluctuation analysis; SampEn, sample entropy.

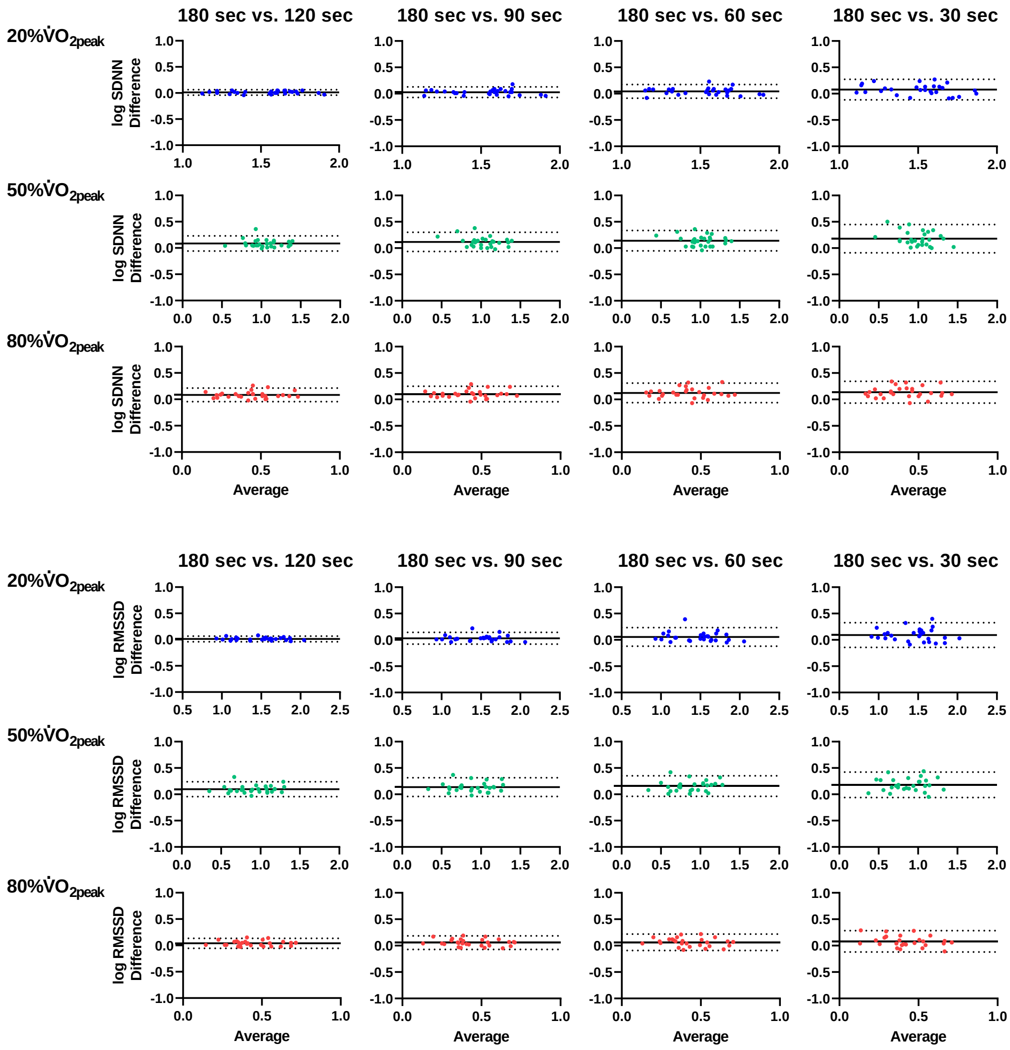

Figure S1. Bland-Altman plots between the 180 sec and other ultra-short-term (120 sec, 90 sec, 60 sec, and 30 sec) HRVs in time-domain. SDNN, standard deviation of all RR intervals; RMSSD, root mean square of successive RR interval differences; log, logarithmic; sec, seconds; $\dot{V}O_{2peak}$, peak oxygen uptake. The solid lines indicate the mean difference between the two values. Dash lines are the 95% limits of agreement.

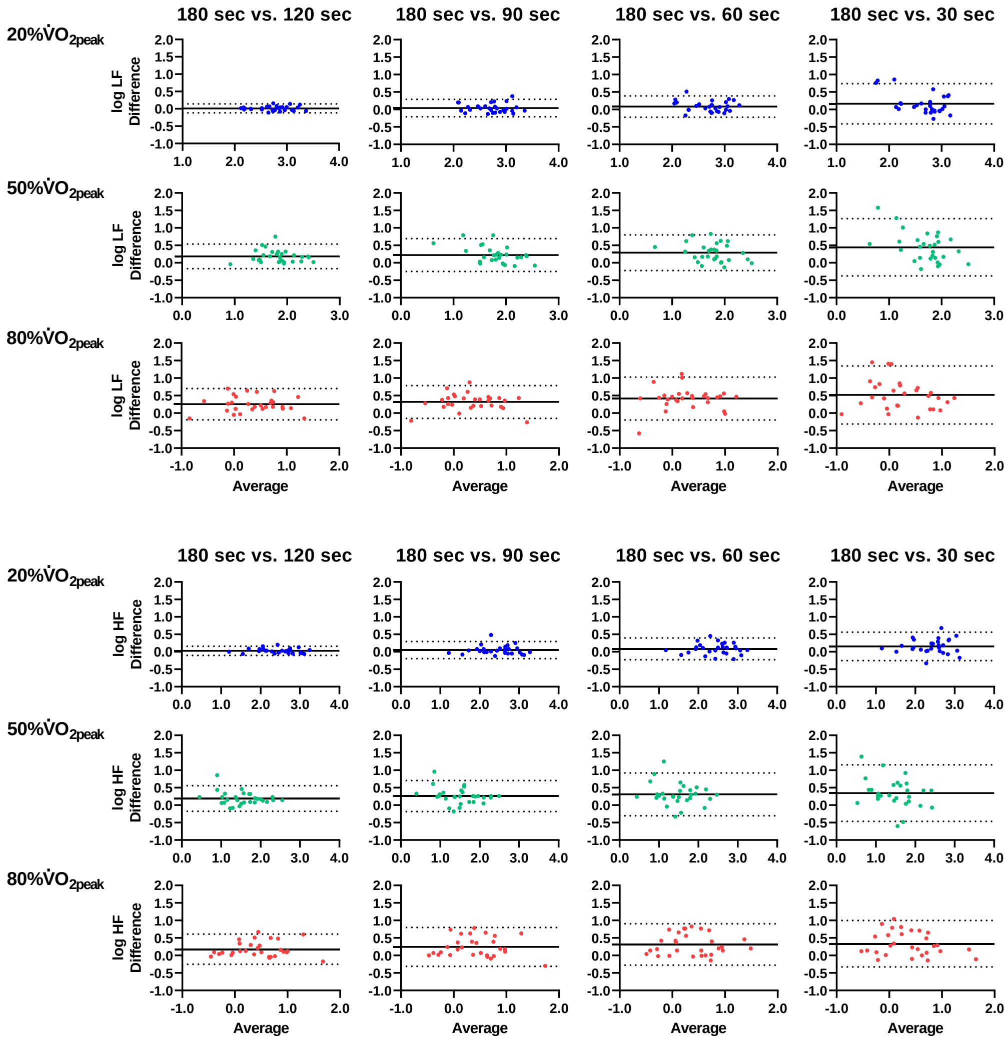

Figure S2. Bland-Altman plots between the 180 sec and other ultra-short-term (120 sec, 90 sec, 60 sec, and 30 sec) HRVs in frequency-domain. LF, low-frequency power; HF, high-frequency power; log, logarithmic; sec, seconds; $\dot{V}O_{2peak}$, peak oxygen uptake. The solid lines indicate the mean difference between the two values. Dash lines are the 95% limits of agreement.

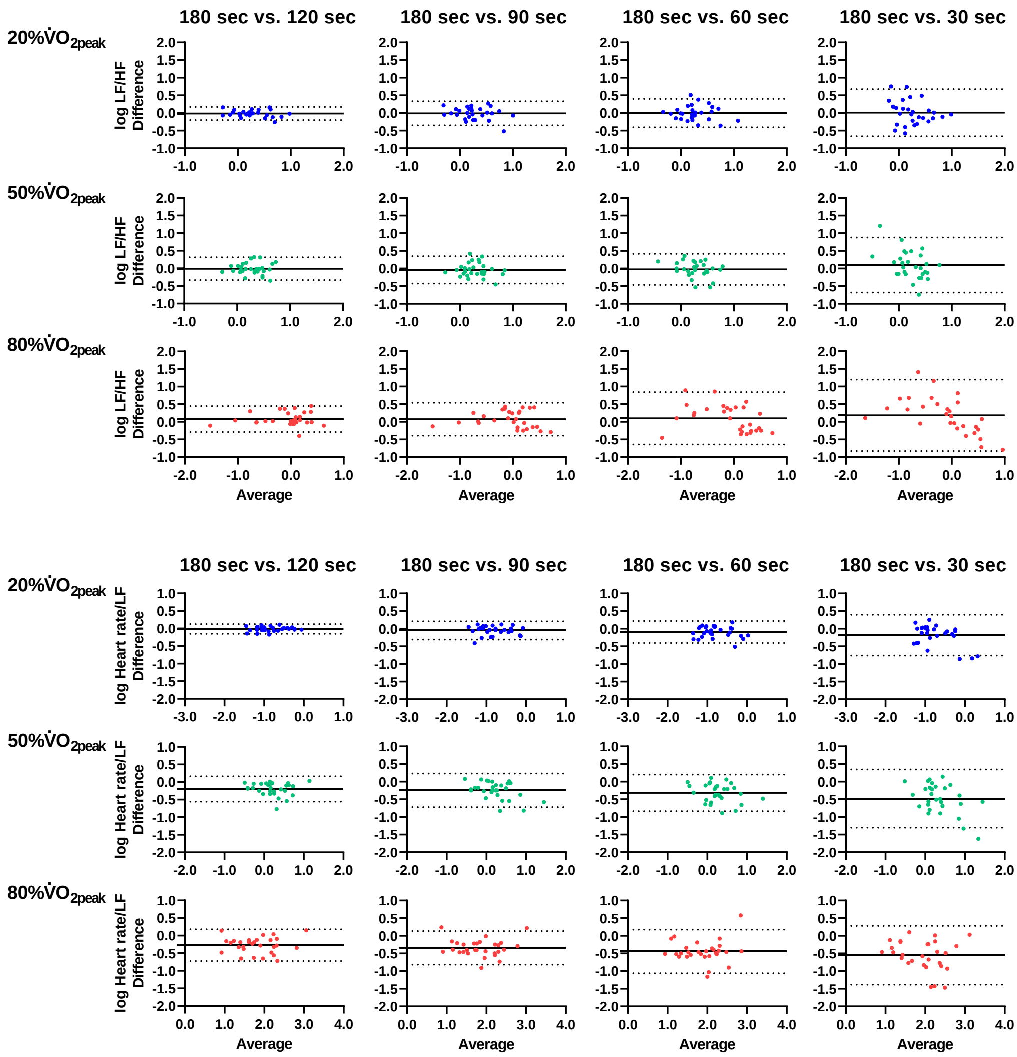

Figure S3. Bland-Altman plots between the 180 sec and other ultra-short-term (120 sec, 90 sec, 60 sec, and 30 sec) HRVs in the frequency-domain analysis. LF, low-frequency power; HF, high-frequency power; log, logarithmic; sec, seconds; $\dot{V}O_{2peak}$, peak oxygen uptake. The solid lines indicate the mean difference between the two values. Dash lines are the 95% limits of agreement.

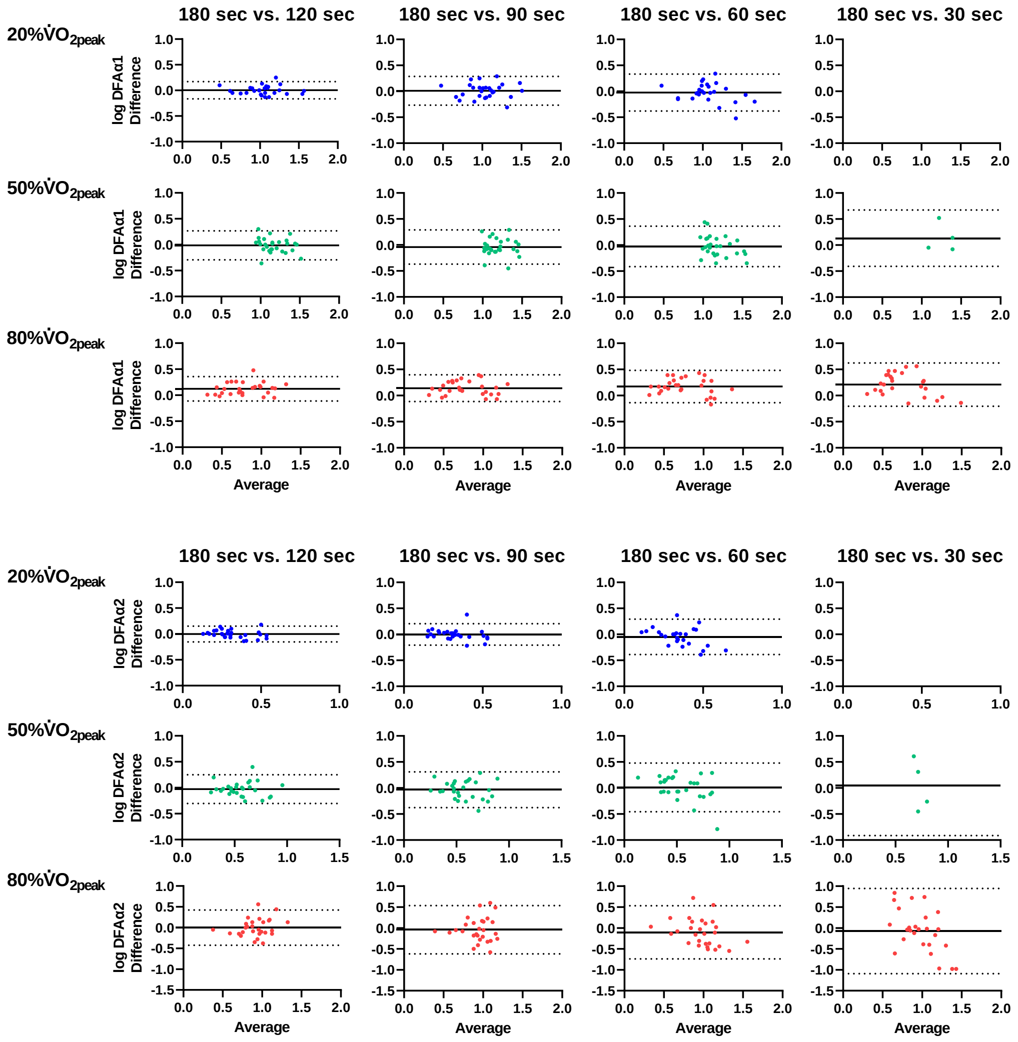

Figure S4. Bland-Altman plots between the 180 sec and other ultra-short-term (120 sec, 90 sec, 60 sec, and 30 sec) HRVs in DFA of non-linear analysis. DFA, detrended fluctuation analysis; log, logarithmic; sec, seconds; $\dot{V}O_{2peak}$, peak oxygen uptake. The solid lines indicate the mean difference between the two values. Dash lines are the 95% limits of agreement.

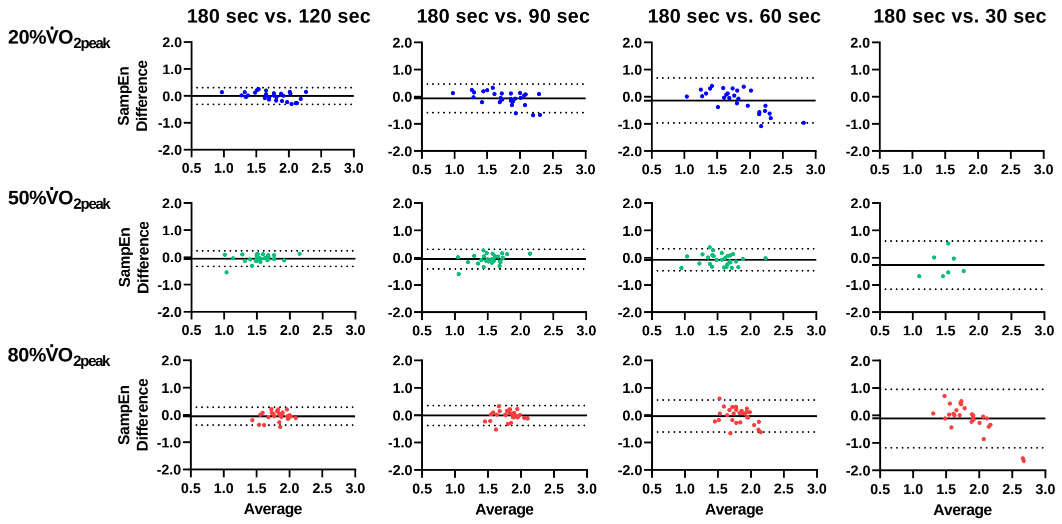

Figure S5. Bland-Altman plots between the 180 sec and other ultra-short-term (120 sec, 90 sec, 60 sec, and 30 sec) HRVs in SampEn of non-linear analysis. SampEn, sample entropy; log, logarithmic; sec, seconds; $\dot{V}O_{2peak}$, peak oxygen uptake. The solid lines indicate the mean difference between the two values. Dash lines are the 95% limits of agreement.
